# Editorial Comment: Single-use flexible ureteroscopes: update and perspective in developing countries. A narrative review

**DOI:** 10.1590/S1677-5538.IBJU.2021.0475.1

**Published:** 2022-04-14

**Authors:** Bruno Marroig

**Affiliations:** 1 Universidade do Estado do Rio de Janeiro Departamento de Cirurgia Geral Rio de Janeiro RJ Brasil Departamento de Cirurgia Geral, Universidade do Estado do Rio de Janeiro - UERJ, Rio de Janeiro, RJ, Brasil

## COMMENT

The article by Mazzucchi et al. presents an excellent review of the characteristics of flexible, optical or digital ureteroscopes, single-use or not, in addition to describing the advantages and disadvantages of each. As an additional objective, this article contains a benefit-cost analysis between the different devices (1).

When analyzing the costs of devices and procedures in developing countries, one needs to keep in mind the availability of resources, sometimes present only in larger cities, and the real advantage of opting for the higher-cost alternative, such as digital single-use ureteroscopes.

In Brazil, there are two distinct realities: private practice medicine and public health medicine. In public health settings, the cost of disposable or reusable ureteroscopes from a single institution can be accounted for by the institution, even though the public system often cannot afford adequate equipment maintenance programs. Conversely, in private hospitals, where several providers and health insurance companies are involved in the process, a cost-benefit analysis becomes more difficult. Reusable ureteroscopes are often owned by the supplier of disposable materials, such as fiber lasers, baskets, and catheters. The cost to the health insurance company will be higher in case of surgery where the disposable ureteroscope is used in addition to other devices, as the company does not need to pay for the reusable ureteroscopes.

Regarding the performance of ureteroscopes, there is no sufficient technical data that is consistent to determine that disposable devices are superior to reusable ureteroscopes. In general, optical reusable ureteroscopes display a smaller outer diameter of the insertion tube than the digital ones. An example relies on the most used ureteroscopes, the Storz FlexX2, with 7.5 Fr, whereas the digital ones have around 9.0 Fr (8.7 to 9.9 Fr). This may cause difficulties in thinner ureters or in those subjects without the previous presence of double j catheter (2).

Complex stones, especially in the lower pole, require greater deflection of the ureteroscope. A previous study investigated the access to the most caudal calyx of the lower caliceal group with the optical ureteroscope and showed that, depending on the anatomy of the collecting system, the access rate varies between 64 and 85% of cases (3). In these cases, when there is a greater risk of damage to the device due to forced deflection, the adoption of a single-use ureteroscope is indicated.

Previous evidence showed that surgery time is reduced by up to 30% when disposable devices are used (4–6). We can infer that the use of these devices can be advantageous for large stones (> 2 cm in the largest diameter) as the stone mass will require more time to be fragmented, and surgeries with operative time longer than 90 min significantly increase the risk of infection (7).

The stone-free rates are higher or similar when using the disposable ureteroscope according to several authors (6, 8) but not all authors report the same conclusion. Mager et al. compared the use of disposable and reusable ureteroscopes and reported similar stone-free rate, operative time and fluoroscopy time, and a higher rate of complications when using the single-use device (9).

In their article, Mazzucchi et al. perfectly show different situations where we observe advantages and disadvantages of using each type of device and the different results found by different authors (1).

The use of the material by several surgeons, especially those with less experience, is known to increase the frequency of damage to the devices, thereby increasing the associated costs. In hospital units where there is medical residency, these occurrences must be anticipated and included in the expected costs per procedure. After the learning curve, however, it is expected that the surgeon’s experience will result in better clinical outcomes, with shorter operative time and hospital stay, lower complication rates and higher stone-free rates.

When initially marketed in the 1990s, the Holmium-Yag laser was expensive and hardly available in both public and even in private hospitals, as its authorization by health insurance companies was quite restricted. Currently, usage of laser is already a reality in many health services. As we monitor the evolution of laser usage, we believe that the cost of single-use ureteroscopes will decrease over time and that they will be more readily available for use in health settings.
